# Sex, not gender. A plea for accuracy

**DOI:** 10.1038/s12276-019-0341-0

**Published:** 2019-11-08

**Authors:** Cristina Richie

**Affiliations:** Brody School of Medicine, 600 Moye Boulevard, East Carolina University, Greenville, NC 27834 USA

**Keywords:** Health care, Medical research

A recent *Experimental & Molecular Medicine* article contained an internally inaccurate title: “Gender-independent efficacy of mesenchymal stem cell therapy in sex hormone-deficient bone loss via immunosuppression and resident stem cell recovery”^1^. The title should have read “*Sex*-independent efficacy of mesenchymal stem cell therapy in sex hormone-deficient bone loss via immunosuppression and resident stem cell recovery”. Sex is biological. It is determined by the X and Y chromosomes. The article focused on male and female mice. There is no clinical identifier for gender. Thus, “sex” should have been used in the title and throughout the article.

Gender refers to societal expectations regarding human male and female appearance, behavior, interests, and lifestyle. Gender is contrived and then imposed by hegemonic structures highly inflected by geography, age bracket, income, race, religion, era, and education level. Although the article mentions “two genders,” society recognizes a plurality of genders, for instance, cisgender, transgender, genderplural, gender ambiguous, and genderqueer. In contrast, science recognizes two sexes—male and female—with intersex conditions as mutational variations^2^.

The conflation of sex and gender is problematic in several ways. First, it leads to inaccuracies. In one place, the authors erroneously used the term “gender”, although the reference note pertained to sex. Second, the authors failed to define or differentiate between sex and gender; instead, they used the terms interchangeably throughout the article, thus significantly changing the implications of the findings. Third, if the study had actually investigated “gender”-independent efficacy, the design would have included a questionnaire regarding the participant’s perceived or chosen gender (i.e., whether the participants felt more masculine, feminine, neither, or both) instead of using a simple sex determination of male or female. Of course, mice do not have genders.

In the scientific world, confusing gender and sex reifies—instead of correcting—inaccurate language and encourages others to do so by replication. In the human world, confusing gender and sex damages people who reject gender norms. The classification of gender dysphoria as a mental disorder in the Diagnostic and Statistical Manual of Mental Disorders (DSM) attests to this.

The second wave of feminism in the United States began using the term “gender” to rightly name the systemic sexism that resulted in women getting paid less than men, excluded women from male-dominated vocations, and prevented women from entering male-only institutions based on stereotypes about women. Eventually, the term “sex” fell into disuse, and “gender” became the standard^3^. High-ranking journals have not been immune to this trend, which is largely influenced by American feminists and not by international scientists. However, the absence of a clear demarcation between sex and gender compromises accuracy while also undermining the credibility of the scientific journals that publish these studies.

I am making an open plea for science and medicine to return to linguistic precision, scientific integrity, and authorial accountability.

## References

[1] Sui BD (2018). Gender-independent efficacy of mesenchymal stem cell therapy in sex hormone-deficient bone loss via immunosuppression and resident stem cell recovery. Exp. Mol. Med..

[2] Editorial. US proposal for defining gender has no basis in science. *Nature* **563**10.1038/d41586-018-07238-8. (2018). 10.1038/d41586-018-07238-8 30377332

[3] Hausman BL (1998). Sex before gender: Charlotte Perkins Gilman and the evolutionary paradigm of utopia. Feminist Stud..

